# Assessment of Community-Level Vulnerability and Access to Medications for Opioid Use Disorder

**DOI:** 10.1001/jamanetworkopen.2022.7028

**Published:** 2022-04-19

**Authors:** Paul J. Joudrey, Marynia Kolak, Qinyun Lin, Susan Paykin, Vidal Anguiano, Emily A. Wang

**Affiliations:** 1 Program in Addiction Medicine, Department of Internal Medicine, Yale School of Medicine, New Haven, Connecticut; 2 Healthy Regions & Policies Lab, Center for Spatial Data Science, University of Chicago, Chicago, Illinois; 3 SEICHE Center for Health and Justice, Department of Internal Medicine, Yale School of Medicine, New Haven, Connecticut

## Abstract

**Importance:**

Given that COVID-19 and recent natural disasters exacerbated the shortage of medication for opioid use disorder (MOUD) services and were associated with increased opioid overdose mortality, it is important to examine how a community’s ability to respond to natural disasters and infectious disease outbreaks is associated with MOUD access.

**Objective:**

To examine the association of community vulnerability to disasters and pandemics with geographic access to each of the 3 MOUDs and whether this association differs by urban, suburban, or rural classification.

**Design, Setting, and Participants:**

This cross-sectional study of zip code tabulation areas (ZCTAs) in the continental United States excluding Washington, DC, conducted a geospatial analysis of 2020 treatment location data.

**Exposures:**

Social vulnerability index (US Centers for Disease Control and Prevention measure of vulnerability to disasters or pandemics).

**Main Outcomes and Measures:**

Drive time in minutes from the population-weighted center of the ZCTA to the ZCTA of the nearest treatment location for each treatment type (buprenorphine, methadone, and extended-release naltrexone).

**Results:**

Among 32 604 ZCTAs within the continental US, 170 within Washington, DC, and 20 without an urban-rural classification were excluded, resulting in a final sample of 32 434 ZCTAs. Greater social vulnerability was correlated with longer drive times for methadone (correlation, 0.10; 95% CI, 0.09 to 0.11), but it was not correlated with access to other MOUDs. Among rural ZCTAs, increasing social vulnerability was correlated with shorter drive times to buprenorphine (correlation, –0.10; 95% CI, –0.12 to –0.08) but vulnerability was not correlated with other measures of access. Among suburban ZCTAs, greater vulnerability was correlated with both longer drive times to methadone (correlation, 0.22; 95% CI, 0.20 to 0.24) and extended-release naltrexone (correlation, 0.15; 95% CI, 0.13 to 0.17).

**Conclusions and Relevance:**

In this study, communities with greater vulnerability did not have greater geographic access to MOUD, and the mismatch between vulnerability and medication access was greatest in suburban communities. Rural communities had poor geographic access regardless of vulnerability status. Future disaster preparedness planning should match the location of services to communities with greater vulnerability to prevent inequities in overdose deaths.

## Key Points

**Question:**

Does the association of community vulnerability to disasters and pandemics with access to medications for opioid use disorder vary across urban and rural communities?

**Findings:**

In this cross-sectional study of 32 604 zip codes tabulation area across the continental United States, communities with greater social vulnerability did not have greater geographic access to medications, and the mismatch was greatest in suburban communities. Rural communities had poor geographic access regardless of vulnerability.

**Meaning:**

These findings suggest that disaster preparedness planning should include anticipation of access to medications for opioid use disorder and better match the location of services to communities with greater vulnerability to prevent inequities in opioid overdose deaths.

## Teaser

This cross-sectional study examines the association of community vulnerability to disasters and pandemics with geographic access to 3 medications for opioid use disorder (buprenorphine, methadone, and extended-release naltrexone) and whether this association differs by urban, suburban, or rural classification.

## Introduction

US opioid overdose deaths increased within both urban and rural communities and continue to rise in the context of the COVID-19 pandemic.^1,2^ Treatment with the 3 US Food and Drug Administration–approved medications for opioid use disorder (MOUDs), buprenorphine, methadone, and extended-release naltrexone, can reduce opioid overdose deaths,^3,4,5^ but they are not equivalent or interchangeable.^6^ Buprenorphine is a partial opioid agonist available within office-based settings via Drug Addiction Treatment Act of 2000 (DATA 2000) waivers.^6,7^ Methadone is a full opioid agonist but can only be provisioned at federally certified opioid treatment programs (OTPs).^6,7^ Extended-release naltrexone is an opioid antagonist and is typically dispensed by a specialty pharmacy and administered by the prescriber.^6,7^ Research suggests there is variation in patient preference for these 3 medications, and historical, socioeconomic, racial, and other structural factors affect the availability and perceptions of MOUD services.^8,9,10,11^ Given the differences in pharmacology, delivery, and patient preference, the 3 MOUDs should be accessible in all communities to facilitate treatment individualization and maximization of retention.^6^ However, there is a shortage of MOUD services, disproportionately affecting rural communities, and differences in availability by race, contributing to racial inequities in access.^12,13^ Most US patients with opioid use disorder (OUD) never initiate an MOUD because of factors such as cost, stigma of medication treatment, acceptability of services, and geographic availability.^6,14^

Evidence suggests COVID-19 and recent natural disasters have exacerbated the shortage of MOUD services^15,16,17,18,19^ and were associated with increased opioid overdose and chronic disease mortality.^2,16,18,20^ In the context of COVID-19, US opioid overdose deaths increased 29% from November 2019 to November 2020.^2^ Despite efforts to mitigate the impact of the pandemic on MOUD services, such as increased telemedicine and take-home methadone dosing,^19,21^ there was a reduction in locations accepting new patients initiating methadone and long wait times to initiate a medication.^19^

With the ongoing COVID-19 pandemic and the expected increased frequency of climate change–related extreme weather events,^22,23^ it is important to examine how a community’s ability to respond to disasters and infectious disease outbreaks is associated with current access to MOUDs, especially given the already uneven access to the medications. The World Health Organization and the US Substance Abuse and Mental Health Services Administration (SAMHSA) have recommended state and local agencies develop disaster plans for patients receiving methadone and buprenorphine.^24,25,26^ The recommendations include planning for the transfer of patients to nearby facilities in the case of closure, but the feasibility of this option depends on available nearby facilities. To our knowledge, no past studies have examined the association between access to each medication and a community’s ability to respond to disaster or disease outbreaks. Identifying communities with greater vulnerability to disasters and low access to MOUDs could inform interventions and policies aiming to expand MOUD access and mitigate future disparities in mortality. Therefore, we examined the association between social vulnerability and access to each of the 3 MOUDs within the continental US and whether this association was affected by community urban-rural classification.

## Methods

### Study Overview and Data Sources

We completed a cross-sectional geospatial analysis within the continental US and followed the Strengthening the Reporting of Observational Studies in Epidemiology (STROBE) reporting guideline for cross-sectional studies. The University of Chicago institutional review board determined this study exempt from review and the requirement for informed consent because it did not involve human participants. We obtained 2018 census tract social vulnerability index (SVI) data from the US Centers for Disease Control and Prevention.^27^ The SVI is a validated measure of community vulnerability to natural (eg, hurricane or infectious disease) or human-caused (eg, chemical spill) stressors.^28,29,30,31^ The SVI measures overall vulnerability and vulnerability across 4 specific themes: (1) socioeconomic status, (2) household composition and disability, (3) racial and ethnic minority status and language, and (4) housing type and transportation.^27^ The SVI assigns each tract a score based on percentile rank (scored 0 to 1, with 1 representing the highest vulnerability) (eMethods 1 in the Supplement).^27^

We measured geographic access to the 3 MOUDs using the following data sources. The primary data source was the 2020 SAMHSA Behavioral Health Treatment Services Locator for all substance use treatment clinics providing methadone and extended-release naltrexone and DATA 2000 waiver buprenorphine providers.^32^ Also, because extended-release naltrexone may be provisioned outside of substance use treatment clinics, we obtained location data on all clinicians registered with the pharmaceutical manufacturer as providing extended-release naltrexone from the website on August 29, 2020.^33^ To provide a comparison of accessing treatment services for another chronic disease necessitating thrice weekly visits, we obtained dialysis center location data from the US Centers for Medicare & Medicaid Services’ Dialysis Facility Compare database on May 12, 2020.^34^

### Study Population

We included all zip code tabulation areas (ZCTAs) within the continental US. ZCTAs are generalized aerial representations of populated US Postal Service zip code service areas created by the Census Bureau.^28^ We used ZCTAs because they are often the smallest geographic unit available to health researchers. We excluded Washington, DC ZCTAs because the travel cost matrix used for drive time estimation only included US states.

### Dependent Variable

Our primary outcome was drive time in minutes from the population-weighted center of the ZCTA to the ZCTA of the nearest treatment location for each treatment type: buprenorphine, methadone, extended-release naltrexone, and dialysis. We excluded treatment locations outside the continental US. We excluded any treatment location without an assigned ZCTA (eAppendix 1 in the Supplement). To calculate access outcomes nationally, we generated an origin-destination matrix of travel times along the street network using Open Source Routing Machine to ZCTAs within 100 km.^35^ To calculate drive time to the nearest treatment location, we used the Python spatial_access package,^35^ using the travel time matrix. For origin ZCTAs also containing the treatment destination, the drive time was estimated as 0 minutes.

Secondary outcomes were the number of treatment locations within or near a ZCTA and number of available MOUD types (0-3). Treatment locations within a 30-minute drive time of the population-weighted center of the ZCTA for each treatment type were tabulated with the spatial_access package.^35^ We used a 30-minute threshold to represent the number of services available in a ZCTA because 30 minutes is a widely accepted standard for acceptable geographic access for Medicaid beneficiaries and has been used to examine access to methadone for people with OUD and dialysis for people with end-stage kidney disease (ESKD).^36,37,38,39^ We also created a count of treatment locations within a 30-minute drive time per 100 000 adults between ages 18 and 64 years for each ZCTA based on the American Community Survey 2018 5-year estimate.

### Independent Variable

Consistent with previous studies,^40^ we converted census tract SVI scores into zip code–scale scores using the US Department of Housing and Urban Development Zip Code Crosswalk.^41^ When a zip code spanned multiple census tracts, we calculated weighted average SVI scores using the ratio between the total addresses in each tract over the total addresses in the entire zip code area.^42^ We matched all zip codes with SVI scores to their assigned ZCTAs.

### Covariates

Covariates included zip codes classification by urbanicity. We obtained 2010 Rural-Urban Commuting Area (RUCA) codes for zip codes from the US Department of Agriculture and University of Washington.^43^ We classified zip codes as either urban (codes 1 and 1.1), suburban (codes 2, 2.1, 4, and 4.1), or rural (all other codes) using RUCA codes (eMethods 2 in the Supplement).^44,45^ Based on past public health literature,^46^ we treat urbanicity as an effect modifier, reflective of the context within which the association between SVI and MOUD access occurs.

### Statistical Analysis

First, we identified the count of ZCTAs, total population, total population between the ages of 18 and 64 years, and treatment locations within the continental US as well as the median SVI score among all ZCTAs and within each urban-rural strata. Then we used a Kruskal-Wallis test for comparisons of drive time to the 4 treatment types and for comparisons by urban-rural strata.

We created correlation matrices using Spearman rank correlation among all ZCTAs and among each urban-rural strata to examine the association of overall SVI and each SVI theme with access to each treatment type. For each correlation matrix, we reversed the direction of the count of treatment locations within a 30-minute drive time so that the direction of correlation was aligned with drive time. We used a Bonferroni correction for multiple comparisons. Given our large sample size, all hypothesis tests were 2-sided with α = .001. We reported correlations of magnitude between –0.09 and 0.09 as no correlation regardless of statistical significance. We used pairwise deletion for missing SVI and primary or secondary outcome data. We completed our analyses in the R software environment version 4.0.2 (R Project for Statistical Computing).

## Results

Among 32 604 ZCTAs within the continental US, we excluded 170 within Washington, DC, resulting in a sample of 32 434 ZCTAs. For analyses stratified by urban-rural classification, we excluded 20 ZCTAs without a RUCA code. Among 32 584 ZCTAs with a RUCA code, 10 657 (33%) were urban, 8067 (25%) were suburban, and 13 860 (43%) were rural (eTable 1 and eFigure in the Supplement). Of the more than 198 million individuals between age 18 and 64 years in ZCTAs with a RUCA code, 76% lived in urban, 15% lived in suburban, and 9% lived in rural ZCTAs. Median overall and theme SVI scores increased (ie, indicated more vulnerability) with increasing rural ZCTA classification except for vulnerability owing to racial and ethnic minority status and language, which decreased with increasing rural ZCTA classification (eTable 2 in the Supplement). Less than 1% of ZCTAs were excluded due to a missing SVI value.

**Figure 1.**
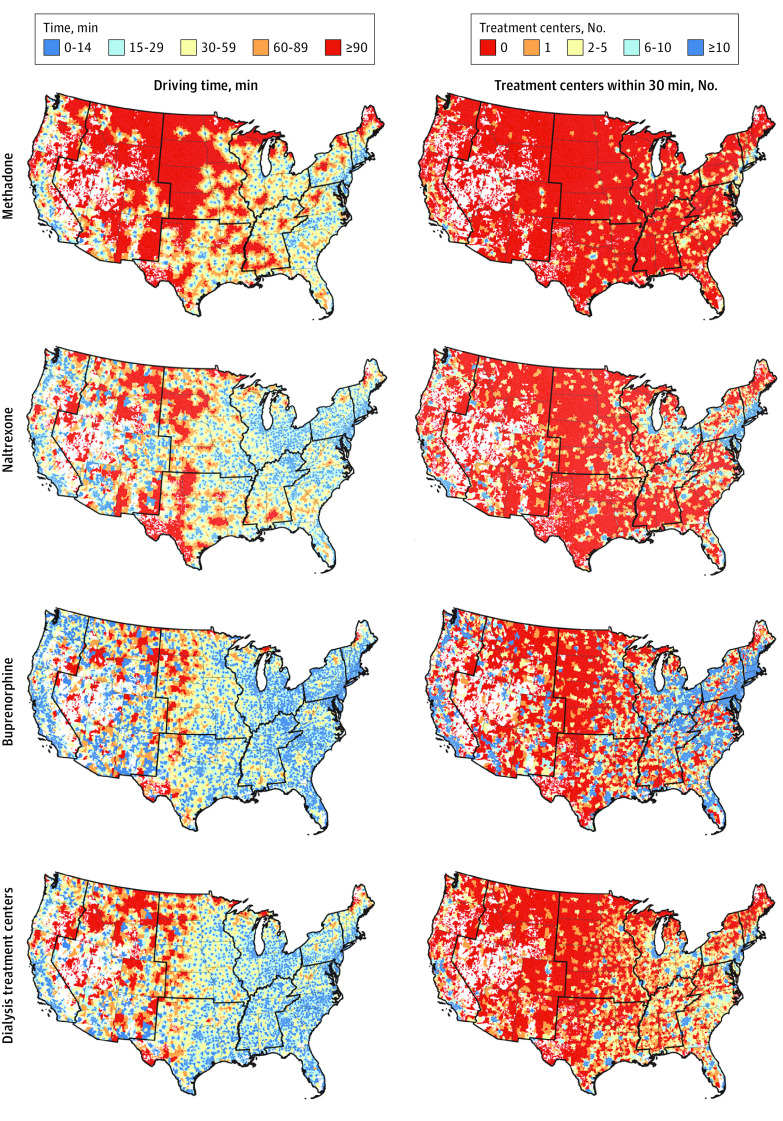
Geographic Access to Treatment Locations Among Zip Codes Within the Continental United States in 2020 Drive time represents the time in minutes from the population weighted center of the zip code tabulation area to the zip code tabulation area of the nearest treatment location for each treatment type. Count within 30 minutes represents the number of treatment locations within a 30-minute drive of a zip code tabulation area. Unpopulated large land areas, such as national parks or large water bodies, are not assigned to a ZCTA, and these gaps are shown in white.

**Figure 2.**
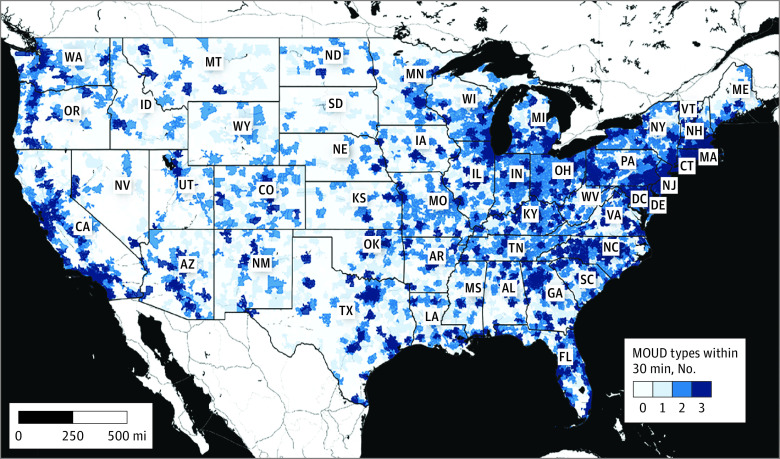
Count of Medications for Opioid Use Disorder Types Within a 30-Minute Drive of Every Population-Weighted Zip Code Tabulation Area Centroid in the Continental United States in 2020 Total number of medications for opioid use disorder (MOUD) treatment types (0 to 3) within a 30-minute drive of the population weighted center of the zip code tabulation area. Basemap created by Stamen Toner, World Geodetic System, 1984.

Among all ZCTAs, median (IQR) drive time to the nearest treatment location was greatest for methadone (35 [16-60] minutes) and shortest for buprenorphine (16 [0-30] minutes; *P* < .001). Only the median drive time to buprenorphine was shorter than median drive time to dialysis (20 [8-34] minutes). For all treatment types, median drive time increased with increasing rural ZCTA classification (Table 1 and Figure 1). For all treatment types, the median count of treatment locations within a 30-minute drive time decreased with increasing rural ZCTA classification. These results were unchanged after accounting for the number of adults within ZCTAs between the ages of 18 and 64 years. Among all ZCTAs, 152 090 242 (77%) adults aged 18 to 64 years lived in a ZCTA with all 3 MOUD within a 30-minute drive (Figure 2).

**Table 1.** Geographic Access to Treatment Services Among ZCTAs Within the Continental United States in 2020

Measure of access	ZCTA, Median (IQR)				*P* value^a^
	Overall (N = 32 584)	Rural (n = 13 860)	Suburban (n = 8067)	Urban (n = 10 657)	
**Median drive time, min** ^b^					
Methadone	35 (16-60)	62 (44-84)	39 (27-54)	13 (7-20)	<.001
Extended-release naltrexone	22 (9-40)	38 (23-59)	25 (16-37)	7 (0-12)	<.001
Dialysis	20 (8-34)	33 (22-49)	22 (15-32)	6 (0-11)	<.001
Buprenorphine	16 (0-30)	28 (17-43)	19 (10-28)	0 (0-7)	<.001
**Locations within 30 min** ^c^					
Methadone	0 (0-2)	0 (0-0)	0 (0-1)	3 (1-8)	<.001
Extended-release naltrexone	2 (0-10)	0 (0-1)	1 (0-4)	20 (8-42)	<.001
Dialysis	2 (0-8)	0 (0-1)	2 (0-3)	16 (7-36)	<.001
Buprenorphine	8 (1-53)	1 (0-5)	6 (1-19)	117 (43-269)	<.001
**Locations per 100 000 population** ^d^					
Methadone	0 (0-31)	0 (0-0)	0 (0-20)	32 (11-99)	<.001
Extended-release naltrexone	62 (0-302)	0 (0-118)	44 (0-272)	202 (72-544)	<.001
Dialysis	69 (0-253)	0 (0-128)	62 (0-241)	163 (67-427)	<.001
Buprenorphine	379 (24-1619)	78 (0-587)	287 (32-1304)	1212 (441-3468)	<.001

Abbreviations: ZCTA, zip code tabulation area.

^a^ Rural, suburban, and urban results were compared using a Kruskal-Wallis rank sum test.

^b^ Drive time represents the time in minutes from the population weighted center of the ZCTA to the ZCTA of the nearest treatment location for each treatment type.

^c^ Locations within 30 minutes represents the number of treatment locations within 30-minute drive of a ZCTA.

^d^ Locations per 100 000 population refers to the number of treatment locations within a 30-minute drive of a ZCTA per 100 000 zip code inhabitants.

### Social Vulnerability and Treatment Access

**Figure 3.**
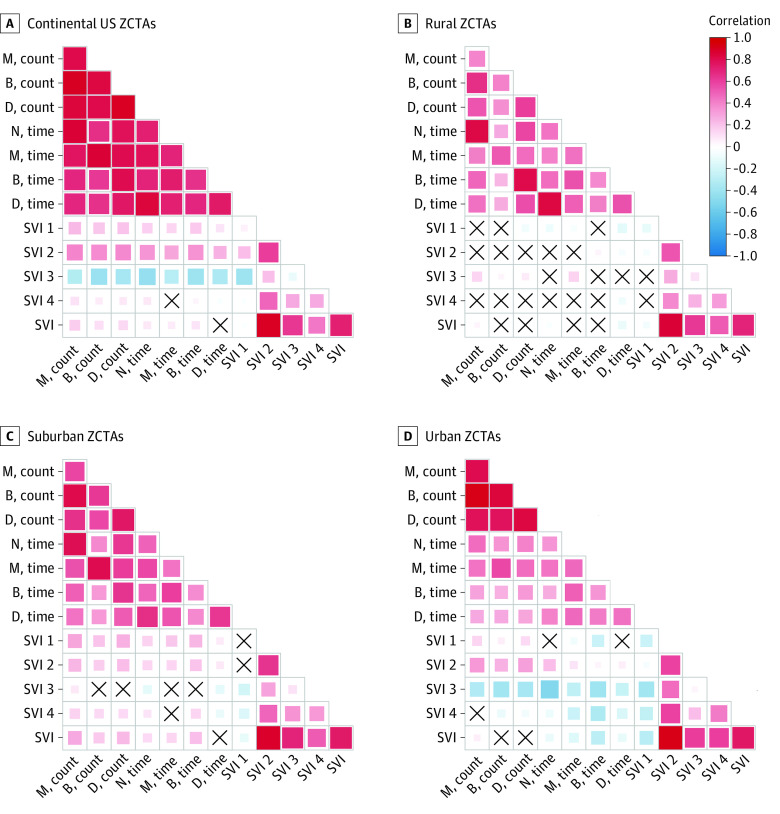
Correlation Between Zip Code Social Vulnerability Index and Geographic Access to Methadone (M), Buprenorphine (B), Dialysis (D), and Extended-Release Naltrexone (N) Treatment Within the Continental United States in 2020 The shading is representative of the magnitude of Spearman rank correlation, with red indicating positive correlations and blue indicating negative correlations. We reversed the direction of all count access metrics so that the directions of correlations are aligned. For correlations between access metrics and social vulnerability index (SVI) scores, positive correlations (in red) indicate more vulnerable zip code tabulation areas (ZCTAs) have worse accessibility (ie, fewer resources or longer drive time), while negative correlations (in blue) indicate more vulnerable zip code areas have better accessibility (ie, more resources or shorter drive time). Boxes marked with an X represents no correlation.

Among all ZCTAs, greater overall social vulnerability was not associated with greater access to MOUD (Figure 3). For methadone, greater overall social vulnerability was correlated with both longer drive times (correlation, 0.10; 95% CI, 0.09-0.11) and less available treatment locations (correlation, 0.11; 95% CI, 0.10-0.13; *P* < .001) compared with other modalities of MOUD treatment. Among all ZCTAs, greater vulnerability owing to racial and ethnic minority status and language was correlated with greater access to MOUD. There was either no correlation or a correlation with less access to MOUD for vulnerability owing to socioeconomic status, household composition and disability, and housing type and transportation. Overall social vulnerability was not associated with greater access to dialysis for ESKD (eAppendix 2 in the Supplement).

The association between social vulnerability and access to all 3 types of MOUD varied depending on urban-rural classification. Among rural ZCTAs, increasing overall social vulnerability was correlated with shorter drive times to buprenorphine (correlation –0.10; 95% CI, –0.12 to –0.08); *P* < .001) but not correlated with other measures of access to MOUD. For vulnerability by theme, vulnerability owing to socioeconomic status was correlated with shorter drive time to buprenorphine (correlation –0.13; 95% CI, –0.15 to –0.11; *P* < .001). Vulnerability owing to racial and ethnic minority status and language was correlated with longer drive time (correlation, 0.16; 95% CI, 0.14-0.17) and less available treatment locations (correlation, 0.15; 95% CI, 0.13-0.16) for extended-release naltrexone (*P* < .001). While drive time to treatment services largely did not vary by SVI among rural ZCTAs, median drive times were longer in rural ZCTAs compared with suburban and urban ZCTAs, including among rural ZCTAs in the highest quartile of SVI (Table 2).

**Table 2.** Median Drive Time in Minutes to Treatment by Social Vulnerability Index Within the Continental US in 2020

Social vulnerability index theme quartiles	Drive time by drug type and rural-urban classification, median (IQR), min^a^											
	Rural				Suburban				Urban			
	M	N	D	B	M	N	D	B	M	N	D	B
Socioeconomic vulnerability												
First	60 (40-84)	40 (24-67)	34.5 (23-56)	31 (19-52)	32 (23-44)	22 (15-31)	22 (16-29)	18 (11-26)	15 (10-21)	8 (0-13)	8 (1.25-13)	0 (0-7)
Second	65 (46-86)	39 (24-61)	35 (23-51)	29 (19-46)	36 (26-49)	23 (16-33)	23 (16-31)	19 (11-27)	13 (8-21)	7 (0-13)	6 (0-12)	0 (0-8)
Third	62 (45-82)	36 (22-55)	32 (22-47)	26 (17-40)	42 (29-57)	25 (15-36.75)	21 (13-31)	19 (7-28)	12 (6-20)	6 (0-13)	5 (0-10)	0 (0-7)
Fourth	61 (43-83)	38 (23-58)	31 (20-45)	25 (15-38)	47 (32-62)	32 (20-46)	23 (13-34)	22 (11-33)	9 (4-15)	6 (0-11)	4 (0-8)	0 (0-6)
Household composition and disability												
First	57 (38-80)	36 (22.5-58)	33 (22-53)	27 (18-42)	33 (23-45)	21 (15-30)	22 (16-29)	17 (11-25)	12 (7-19)	6 (0-11)	7 (0-11)	0 (0-6)
Second	62 (44-83)	39 (24-62)	34 (23-51)	29 (18-46)	37 (26-51)	24 (16-35)	23 (16-31)	19 (11-28)	13 (8-20.25)	7 (0-13)	6 (0-11)	0 (0-7)
Third	63 (44-84)	37 (23-58)	33 (22-48)	28 (18-42)	40 (28-55)	26 (16-39)	22 (13-31)	20 (10-28.5)	13 (7-22)	8 (0-15)	6 (0-11)	0 (0-9)
Fourth	63 (45-84)	39 (24-59)	32 (21-47)	27 (16-41)	45 (32-61)	29 (18-43)	23 (13-34)	21 (10-32)	12 (7-20)	8 (0-14)	6 (0-10)	0 (0-9)
Racial and ethnic minority status and language												
First	61 (42-83)	34 (22-53)	33 (23-48)	27 (18-42)	40 (29-55)	25 (18-36)	24 (17-34)	21 (14-29)	21 (15-29)	14 (9-19)	14 (9-18)	9 (0-14)
Second	61 (44-82)	37 (23-55)	32 (22-47)	27 (17-41)	38 (26-52)	24 (16-34)	23 (16-31)	19 (11-28)	18 (12-26)	11 (5-16)	11 (5-15)	3 (0-11)
Third	63 (46-84)	43 (25-64.75)	32 (21-50)	27 (17-42)	38 (26-53)	26 (15-39)	20 (12-29)	18 (0-28)	14 (9-21)	7 (0-13)	7 (0-12)	0 (0-7)
Fourth	64 (45-86)	49 (30-73)	35 (21-55)	30 (17-49)	38 (26-58)	28 (15-45)	19 (8-28)	17 (0-28.5)	10 (5-15)	5 (0-10)	4 (0-8)	0 (0-4)
Housing type and transportation												
First	61 (44-83)	36 (23-57)	32 (23-47)	28 (19-44)	35 (25-48)	24 (17-34)	24 (18-31)	20 (14-28)	17 (12-23)	10 (5-15)	9 (5-14)	0 (0-10)
Second	61 (43-83)	37 (24-57)	32 (22-46)	28 (18-42)	38 (27-52)	25 (17-36)	23 (16-33)	20 (13-29)	14 (9-21)	7 (0-13)	7 (0-12)	0 (0-7)
Third	62 (43-83)	39 (24-60)	33 (22-50)	28 (17-43)	40 (28-56)	25 (14-39)	21 (12-31)	19 (0-28)	11 (6-18)	5 (0-11)	4 (0-9)	0 (0-5)
Fourth	63 (45-85)	39 (23-61)	34 (22-51)	26 (13.5-42)	45 (30-61)	26 (12-42)	19 (7-30)	16 (0-29)	8 (4-14)	4 (0-9)	4 (0-8)	0 (0-4)

Abbreviations: B, buprenorphine; D, dialysis; M, methadone; N, naltrexone.

^a^ Drive time represents the time in minutes from the population weighted center of the zip code tabulation area to the zip code tabulation area of the nearest treatment location for each treatment type.

Among suburban ZCTAs, greater overall vulnerability was correlated with both longer drive times and less available locations for all MOUDs, except drive time to buprenorphine (no correlation). Greater vulnerability owing to socioeconomic status and household composition and disability were correlated with both longer drive times and less available locations, again with the exception for drive time to buprenorphine. Greater vulnerability due to housing type and transportation was correlated with both longer drive times and less available locations, except for drive time to buprenorphine and extended-release naltrexone. Among suburban ZCTAs, the median (IQR) drive time to methadone increased from the lowest to the highest quartile of vulnerability for socioeconomic status (32 [23-44] minutes to 47 [32-62] minutes), household composition and disability (33 [23-45] minutes to 45 [32-61] minutes), and housing type and transportation (35 [25-48] minutes to 45 [30-61] minutes) (Table 2).

Among urban ZCTAs, greater overall vulnerability was correlated with shorter drive times for all MOUD but not correlated with available treatment locations. Greater vulnerability owing to racial and ethnic minority status and language was correlated with shorter drive times and more available locations for MOUD.

## Discussion

In this cross-sectional geospatial analysis within the continental US, zip codes with greater social vulnerability did not have greater geographic access to each of the 3 MOUDs, showing the degree to which the United States falls short of ensuring equitable access to all MOUDs, especially during natural disasters. Consistent with emerging literature on so-called opioid treatment deserts,^47^ nearly one-quarter of the continental US population lives without access to all 3 MOUDs when using a conservative 30-minute travel threshold. We build on past work by showing that urban-rural inequities were present for most measures of access, including drive time, count of nearby locations, and count of locations per population at risk.^12,48,49^ Drive times were significantly longer for methadone and extended-release naltrexone relative to dialysis centers, despite the prevalence of OUD being greater than that of ESKD.^12,50,51^

A novel finding of this study is that the mismatch between overall social vulnerability and the location of MOUD services was greatest in suburban zip codes compared with rural and urban settings. Ideally, geographic access to MOUD services would be greater within the most vulnerable communities. While the observed correlations were modest in magnitude, we frequently did not find such an association. In examining the specific vulnerability themes, living in suburban communities with lower socioeconomic status or in households with more children, seniors, or individuals with disabilities was associated with less geographic access to methadone and extended-release naltrexone. In contrast, geographic access was largely not associated with social vulnerability in rural zip codes because geographic access was uniformly poor. Living in urban zip codes with greater social vulnerability owing to higher proportion of racial and ethnic minority populations and non-English speakers was associated with greater geographic access to MOUD, suggesting that geographic access may not be as important of a barrier in these communities. Geography is just one dimension of access^52^; MOUD access is also affected by stigma, affordability, accommodation, capacity, and more, and future research should examine these factors by social vulnerability.

We improved on previous research by using 2 measures of small area (ZCTA) geographic access (drive time and count of near locations) to all 3 types of MOUD, while incorporating 2 sources of extended-release naltrexone location data. Our results are consistent with research showing communities with lower socioeconomic status have less geographic access to methadone and buprenorphine,^49^ and we extend these findings to extended-release naltrexone. By examining the 4 SVI themes, we found that communities with social vulnerability owing to housing type and transportation also did not have greater geographic access to MOUDs. While this was also true for vulnerability owing to households with children, older individuals, and individuals with disabilities, its relevance for OUD is less clear because the prevalence of OUD is both lower (ie, among older individuals) and higher (ie, among individuals with disabilities preventing entry into the workforce) among subpopulations within this theme.

Our results call into question current disaster preparedness of OUD services and indicate the need for proactive measures to increase services within communities vulnerable to disasters. Expanding OUD services is especially important in vulnerable suburban areas and across rural communities. Methadone should be a priority given the sizeable barrier to ensuring access across all communities even when stratified by urbanicity. Continuing changes in MOUD services during COVID-19, such as increased methadone take-home allowances, present opportunities to modify the reach of services during disasters.^53^ Furthermore, reducing restrictions on medication units (OTP-affiliated satellite locations for methadone administration and dispensing) and the recent end to the moratorium on new mobile methadone vans may also mitigate urban-rural inequalities and increase MOUD services within communities vulnerable to disaster if strategically implemented.^36,54^ Lastly, allowing methadone treatment outside of OTPs could expand access, but office-based methadone would require federal and state regulatory changes for wide adoption.^55,56^ Currently, SAMHSA, the Drug Enforcement Agency, and State Opioid Treatment Authorities’ disaster planning for methadone prioritize coordination among existing OTPs.^57^ But coordination alone in event of a disaster has been insufficient to ensure access to methadone,^19^ especially in communities without a nearby alternate OTP. Canada increased the flexibility of its federal and provincial methadone regulations, including allowing methadone in office-based settings and dispensing within pharmacies, resulting in an expansion of methadone treatment services.^58^ More flexible methadone regulation at the federal and state level is likely required if the identified inequities in disaster preparedness are to be mitigated. Other health services may require similar interventions to improve disaster preparedness, given that we observed a mismatch between overall social vulnerability and the location of dialysis services.

### Limitations

This study has several limitations. First, this study used the general population between the ages of 18 to 64 years to represent treatment need, and this may differ from the location of individuals with OUD. Second, our measures of geographic access to MOUD did not account for the capacity of treatment locations or whether a location is accepting new patients.^59^ However, our results are consistent with previous research using a gravity model approach, which requires a priori assumptions about the capacity of MOUD treatment locations and patients’ ability to travel, to examine the urban-rural associations between socioeconomic status and access to buprenorphine and methadone.^49^ Third, our secondary outcomes used a 30-minute travel threshold, which likely overestimated availability for people facing transportation barriers. Fourth, our study does not account for mode of transportation or the impact of traffic or weather. Fifth, because drive time was estimated as zero for ZCTAs containing a treatment location, our results likely underestimate drive time, particularly in urban ZCTAs. However, our secondary outcome of number of treatment centers is not affected by this limitation. Sixth, our analysis reflects ZCTA population averages and does not account for variation in social vulnerability within ZCTAs, nor does it represent individual-level access.^60,61^ Seventh, temporal change in vulnerability was beyond the scope of our cross-sectional study but is an important direction for future research.

## Conclusions

In this study, communities within the continental United States with greater social vulnerability did not have greater geographic access to buprenorphine, methadone, or extended-release naltrexone. The mismatch between social vulnerability and the location of MOUD services was greatest in suburban zip codes, but rural zip codes had longer drive times to all 3 MOUDs regardless of vulnerability. MOUD policy and delivery innovations need to address urban-rural inequities and better match the location of services to communities with greater social vulnerability to prevent inequities in opioid overdose deaths during future disasters.

## Notes

Supplement. **eMethods 1.** Social Vulnerability Index **eAppendix 1.** Geocoding of Treatment Locations **eMethods 2.** ZCTA Classification by RUCA Codes **eTable 1.** Location of Treatment Services, Zip Codes, and Zip Code Characteristics by Urbanicity **eFigure 1. **Classification of Zip Code Tabulation Areas by Urbanicity **eFigure 2. **Correlation Between Zip Code Social Vulnerability Index and Geographic Access to Methadone, Buprenorphine, Dialysis, and Extended-Release Naltrexone Treatment Within the Continental US in 2020 **eTable 2.** Zip Code Median Social Vulnerability Index Scores by Urbanicity **eAppendix 2.** Dialysis Treatment and SVI
eReferences.
