# Supplementary material for: Assessment of Community-Level Vulnerability and Access to Medications for Opioid Use Disorder
Source: JAMA Netw Open. 2022 Apr 19;5(4):e227028. doi: 10.1001/jamanetworkopen.2022.7028 (PMC9020217; doi:10.1001/jamanetworkopen.2022.7028)
Supplement: Supplement. — eMethods 1. Social Vulnerability Index eAppendix 1. Geocoding of Treatment Locations eMethods 2. ZCTA Classification by RUCA Codes eTable 1. Location of Treatment Services, Zip Codes, and Zip Code Characteristics by Urbanicity eFigure 1. Classification of Zip Code Tabulation Areas by Urbanicity eFigure 2. Correlation Between Zip Code Social Vulnerability Index and Geographic Access to Methadone, Buprenorphine, Dialysis, and Extended-Release Naltrexone Treatment Within the Continental US in 2020 eTable 2. Zip Code Median Social Vulnerability Index Scores by Urbanicity eAppendix 2. Dialysis Treatment and SVI eReferences. [file jamanetwopen-e227028-s001.pdf]

Supplemental Online Content

Joudrey PJ, Kolak M, Lin Q, Paykin S, Anguiano V Jr, Wang EA. Assessment of community-level vulnerability and access to medications for opioid use disorder. *JAMA Netw Open.* 2022;5(4):e227028. doi:10.1001/jamanetworkopen.2022.7028

eMethods 1. Social Vulnerability Index

eAppendix 1. Geocoding of Treatment Locations

eMethods 2. ZCTA Classification by RUCA Codes

eTable 1. Location of Treatment Services, Zip Codes, and Zip Code Characteristics by Urbanicity

eFigure 1. Classification of Zip Code Tabulation Areas by Urbanicity

eFigure 2. Correlation Between Zip Code Social Vulnerability Index and Geographic Access to Methadone, Buprenorphine, Dialysis, and Extended-Release Naltrexone Treatment Within the Continental US in 2020

eTable 2. Zip Code Median Social Vulnerability Index Scores by Urbanicity

eAppendix 2. Dialysis Treatment and SVI

eReferences.

This supplemental material has been provided by the authors to give readers additional information about their work.

© 2022 Joudrey PJ et al. *JAMA Network Open.*

eMethods 1. Social Vulnerability Index

The SVI is derived from 15 US Census Bureau American Community Survey variables and measures overall vulnerability of a census tract and vulnerability across four specific themes: 1) Socioeconomic status (below poverty, unemployed, income, no high school diploma), 2) Household composition and disability (aged 65 or older, aged 17 or younger, older than age 5 with a disability, single-parent households), 3) Minority status and language (minority, speak English "less than well"), and 4) Housing type and transportation (multi-unit structures, mobile homes, crowding, no vehicle, group quarters such as worker dormitories, skilled nursing facilities, or college dorms).[1] The SVI assigns each tract a score based on percentile rank (scored 0 to 1 with 1 representing the highest vulnerability).[1] The SVI was found to predict disaster related property damage and fatalities over a 12-year period among 10 southeastern states and was included within the inter-agency US Climate Resilience Toolkit to facilitate disaster preparedness planning.[2] In the context of COVID-19, increasing SVI scores were associated with increased community COVID-19 cases and deaths and lower rates of COVID-19 vaccination.[3–5]

© 2022 Joudrey PJ et al. *JAMA Network Open.*

eAppendix 1. Geocoding of Treatment Locations

We matched all buprenorphine (n = 51,191), methadone (n = 1,442), extended-release naltrexone (n = 9,103), and dialysis (n = 7,724) treatment locations to ZCTAs. For analysis by urban-rural classification, we excluded 15 buprenorphine, 2 extended-release naltrexone, and 66 dialysis treatment locations because they resided within the 20 ZCTAs without an assigned RUCA code.

© 2022 Joudrey PJ et al. *JAMA Network Open.*

eMethods 2. ZCTA Classification by RUCA Codes

We modified the widely used University of Washington recommendations for RUCA urban-rural classification by first collapsing the large and small rural codes into one category and then identifying codes 3, 5.1, 7.1, 8.1, and 10.1 as rural instead of suburban. This latter change was driven by observations suggesting the traditional University of Washington approach may overestimate urban and suburban areas. We matched all zip codes assigned a RUCA code to their ZCTA. For analyses stratified by urban-rural classification, we excluded ZCTAs without an assigned RUCA code.

© 2022 Joudrey PJ et al. *JAMA Network Open.*

eTable 1. Location of Treatment Services, Zip Codes, and Zip Code Characteristics by Urbanicity

	Total	Urban	Suburban	Rural
Extended-release naltrexone	9,103	7,004	1,277	820
Methadone	1,442	1,254	145	43
Buprenorphine	51,191	42,784	5,229	3,163
Dialysis	7,724	5,828	1,174	656
ZCTA	32,584	10,657	8,067	13,860
Population	320,026,981	240,888,576	47,938,471	31,199,934
Population 18–64	198,264,579	151,041,785	28,976,347	18,246,447

All zip code tabulation areas (ZCTA) were used for access metric calculation, however, not all ZCTAs are included in the USDA RUCA dataset. Specifically, 20 ZCTAs are missing from the USDA zip code RUCA codes. 0.119% of MOUD and dialysis resources are located in these 20 ZCTAs, thus they were not assigned to any RUCA classification. These include 2 out of 9,103 Naltrexone locations, 15 out of 51,191 Buprenorphine locations and 66 out of 7724 Dialysis locations.

© 2022 Joudrey PJ et al. *JAMA Network Open.*

eFigure 1. Classification of Zip Code Tabulation Areas by Urbanicity

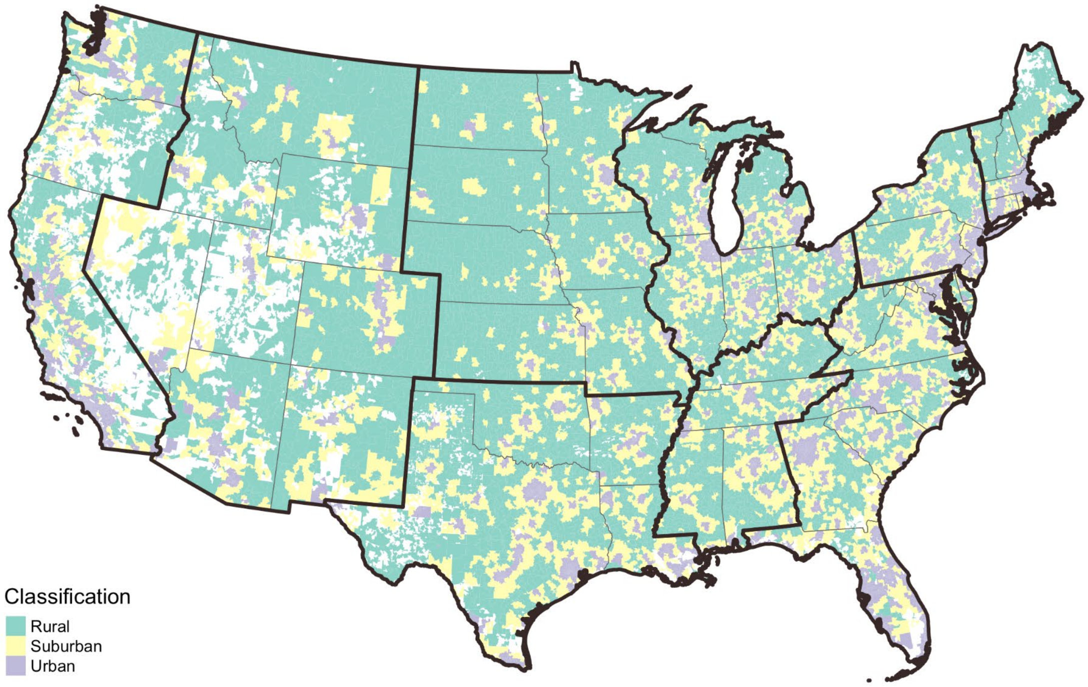

All assigned zip code tabulation areas (ZCTA) in the continental US classified as rural, suburban, or urban, developed based on the U.S. Office of Management and Budget (OMB) RUCA Codes.

© 2022 Joudrey PJ et al. *JAMA Network Open.*

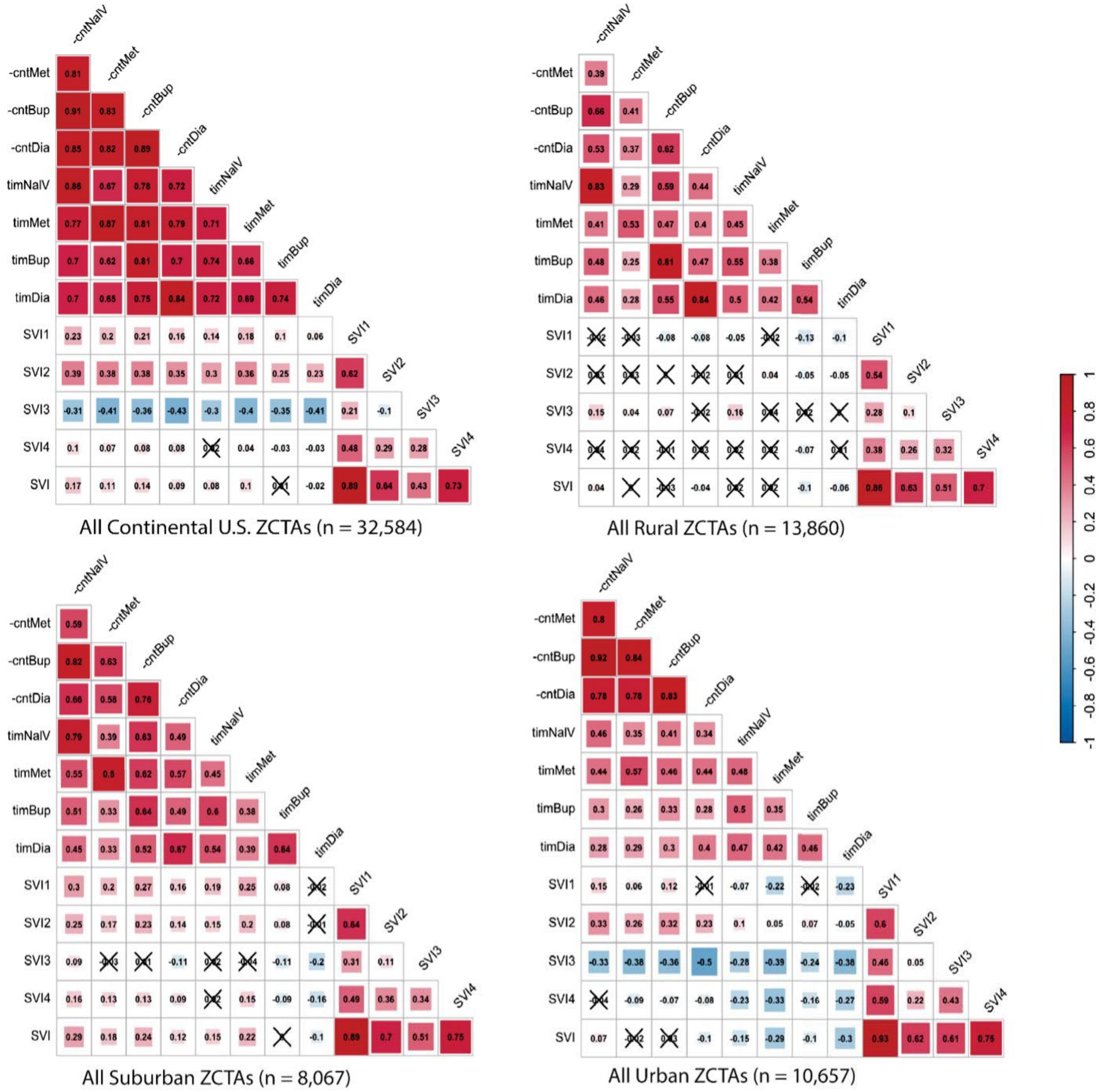

eFigure 2. Correlation Between Zip Code Social Vulnerability Index and Geographic Access to Methadone, Buprenorphine, Dialysis, and Extended-Release Naltrexone Treatment Within the Continental US in 2020

cntMet indicates count of methadone providers within 30 minutes; cntBup, count of buprenorphine providers within 30 minutes; cntDia, count of dialysis providers within 30 minutes; timNalV, driving time to nearest ER-naltrexone provider; timMet, driving time to nearest methadone provider; timBup, driving time to nearest buprenorphine provider; timeDia, driving time to nearest dialysis provider; SVI1, socioeconomic vulnerability (SVI theme 1); SVI2, household composition & disability (SVI theme 2); SVI3, minority status & language (SVI theme 3); SVI4, housing type & transportation (SVI theme 4); SVI, overall vulnerability.

© 2022 Joudrey PJ et al. *JAMA Network Open.*

eTable 2. Zip Code Median Social Vulnerability Index Scores by Urbanicity

Social vulnerability index measure	N	Overall, N = 32,584[1]	Rural, N = 13,860[1]	Suburban, N = 8,067[1]	Urban, N = 10,657	*p*-value
**SVI1: Socioeconomic Status**	32,448	0.50	0.55	0.51	0.41	<0.001
**SVI2: Household Composition & Disability**	32,475	0.59	0.67	0.61	0.42	<0.001
**SVI3: Minority Status & Language**	32,477	0.27	0.18	0.20	0.49	<0.001
**SVI4: Housing Type & Transportation**	32,471	0.50	0.54	0.46	0.48	<0.001
**Overall SVI**	32,445	0.47	0.51	0.45	0.42	<0.001

Source: Authors analyses of 2018 Census tract social vulnerability index (SVI) data from Centers for Disease Control and Prevention. **Notes:** The SVI assigns each zip code tabulation area (ZCTA) a score based on percentile rank (scored 0 to 1 with 1 representing the highest vulnerability). Results presented as a median and compared by urbanicity using a Kruskal-Wallis rank sum test.

© 2022 Joudrey PJ et al. *JAMA Network Open.*

eAppendix 2. Dialysis Treatment and SVI

Greater vulnerability due to household composition and disability was associated with less access to dialysis (correlations 0.23 for drive time 0.35 for available locations, p<0.001), while vulnerability due to minority status and language was associated with greater access (correlations –0.41 for drive time and –0.43 for available locations, p<0.001). Upon stratifying ZCTAs by rural-urban status, within rural ZCTAs there was no correlation between overall social vulnerability and access to dialysis. Among suburban ZCTAs, greater overall vulnerability was associated with shorter drive times and but less available dialysis locations (correlation = –0.10 and 0.12, respectively, p<0.001). Among urban ZCTAs, greater overall vulnerability was associated with shorter drive time times and more available treatment locations (correlation = –0.30 for drive time and –0.10 available locations, p<0.001).

© 2022 Joudrey PJ et al. *JAMA Network Open.*

eReferences.

1. Agency for Toxic Substances and Disease Registry. CDC's Social Vulnerability Index. Place and Health. Published January 19, 2021. Accessed March 10, 2021. https://www.atsdr.cdc.gov/placeandhealth/svi/index.html

2. NOAA's Climate Program Office. US Climate Resilience Toolkit. Published 2021. https://toolkit.climate.gov/tool/social-vulnerability-index

3. Khazanchi R, Beiter ER, Gondi S, Beckman AL, Bilinski A, Ganguli I. County-Level Association of Social Vulnerability with COVID-19 Cases and Deaths in the USA. *J GEN INTERN MED*. 2020;35(9):2784-2787. doi:10.1007/s11606-020-05882-3

4. Karaye IM, Horney JA. The Impact of Social Vulnerability on COVID-19 in the U.S.: An Analysis of Spatially Varying Relationships. *American Journal of Preventive Medicine*. 2020;59(3):317-325. doi:10.1016/j.amepre.2020.06.006

5. Hughes MM, Wang A, Grossman MK, et al. County-Level COVID-19 Vaccination Coverage and Social Vulnerability — United States, December 14, 2020–March 1, 2021. *MMWR Morb Mortal Wkly Rep*. 2021;70(12):431-436. doi:10.15585/mmwr.mm7012e1

© 2022 Joudrey PJ et al. *JAMA Network Open.*
